# Nanoarchitectonics of Ni/CeO_2_ Catalysts: The Effect of Pretreatment on the Low-Temperature Steam Reforming of Glycerol

**DOI:** 10.3390/nano12050816

**Published:** 2022-02-28

**Authors:** Yunzhu Wang, Songshan Zhu, Sufang He, Jichang Lu, Jiangping Liu, Huihui Lu, Di Song, Yongming Luo

**Affiliations:** 1 Faculty of Environmental Science and Engineering, Kunming University of Science and Technology, Kunming 650500, China; wangyunzhu@stu.kust.edu.cn (Y.W.); zhuss525@163.com (S.Z.); lujichangc7@kust.edu.cn (J.L.); liujiangping@gig.ac.cn (J.L.); luhuihuiluhuihui@163.com (H.L.); songdi0512@163.com (D.S.); 2 The Innovation Team for Volatile Organic Compounds Pollutants Control and Resource Utilization of Yunnan Province, Kunming 650500, China; 3 The Higher Educational Key Laboratory for Odorous Volatile Organic Compounds Pollutants Control of Yunnan Province, Kunming 650500, China; 4 Research Center for Analysis and Measurement, Kunming University of Science and Technology, Kunming 650093, China; 5 Faculty of Chemical Engineering, Kunming University of Science and Technology, Kunming 650500, China

**Keywords:** hydrogen production, glycerol steam reforming, direct H_2_ reduction, oxygen vacancies

## Abstract

CeO_2_ nanosphere-supported nickel catalysts were prepared by the wetness impregnation method and employed for hydrogen production from glycerol steam reforming. The dried catalyst precursors were either reduced by H_2_ after thermal calcination or reduced by H_2_ directly without calcination. The catalysts that were reduced by H_2_ without calcination achieved a 95% glycerol conversion at a reaction temperature of only 475 °C, and the catalytic stability was up to 35 h. However, the reaction temperature required of catalysts reduced by H_2_ with calcination was 500 °C, and the catalysts was rapidly inactivated after 25 h of reaction. A series of physicochemical characterization revealed that direct H_2_ reduction without calcination enhanced the concentration of oxygen vacancies. Thus, the nickel dispersion was improved, the nickel nanoparticle size was reduced, and the reduction of nickel was increased. Moreover, the high concentration of oxygen vacancy not only contributed to the increase of H_2_ yield, but also effectively reduced the amount of carbon deposition. The increased active nickel surface area and oxygen vacancies synergistically resulted in the superior catalytic performance for the catalyst that was directly reduced by H_2_ without calcination. The simple, direct hydrogen reduction method remarkably boosts catalytic performance. This strategy can be extended to other supports with redox properties and applied to heterogeneous catalytic reactions involving resistance to sintering and carbon deposition.

## 1. Introduction

The dwindling fossil energy reserves and the environmental problems associated with the development and use of fossil fuel resources have sparked interest in the use of H_2_ as an energy carrier [1]. In addition, considering storage and transportation, it is safer and more convenient to use liquid as the feedstock for producing hydrogen. Among the various raw materials for hydrogen production, glycerol (a biodiesel byproduct) has been regarded as a suitable candidate, which is due to its large mass, large number of hydrogen atoms per molecule, and nontoxicity, as well as its ease of storage and use [2,3,4]. Meanwhile, in the numerous hydrogen production processes, steam reforming of glycerol (GSR) to produce hydrogen has been regarded as a common and efficient process for hydrogen production [5,6].

Based on current research, compared with the high-performance but expensive and scarce noble metals (such as Pt, Pd, and Ir), base metal catalysts, specifically Ni-based catalysts, have been widely researched for hydrogen production in the GSR system, which is due to their excellent properties for breaking bonds (–CH_3_, –CH_2_–, O–H, and –C–C– bonds), as well as their low cost and wide availability [7,8,9,10]. Nevertheless, Ni-based catalysts are easily deactivated, resulting from the agglomeration of nickel nanoparticles and coke deposition [2,11,12,13]. Searching for effective ways to deal with these issues remains a challenge and has attracted a lot of attention in recent years.

For supported metal catalysts, the properties of the supports are critical to the catalytic performance. In addition to affecting the dispersion of the metal and providing support for the metal nanoparticles, the support may also be involved in the catalytic reaction [14,15]. CeO_2_ has been extensively utilized in heterogeneous catalysis because of its distinctive redox behavior and the production of oxygen vacancies by the reduction of Ce^4+^ to Ce^3+^ ions [16,17,18]. According to the previous studie, the oxygen vacancies of CeO_2_can dissociate water and generateabundant surface hydroxyl groups in steam reforming of ethanol, thus increasing the catalytic activity [19]. Furthermore, CeO_2_ is not only used as a redox support to disperse metal nanoparticles but is also used to help oxidize the carbon deposition on the catalyst during the reaction, due to its excellent oxygen storage capacity [20,21]. Pant et al. revealed that a lower coke formation and higher stability were obtained of Ni/CeO_2_ compared to Ni/Al_2_O_3_ catalysts in the steam reforming of glycerol for hydrogen production [22]. For CeO_2_, Ce^4+^ can be reduced to Ce^3+^ in a hydrogen atmosphere, accompanied by the formation of this oxygen vacancy; and oxygen vacancy has the ability to anchor nickel, which can promote nickel dispersion and inhibit nickel aggregation [19]. Hence, increasing the oxygen vacancy concentration by a suitable way may be effective in improving the dispersion of nickel nanoparticles in the catalyst and inhibiting the growth of nickel nanoparticles. Wang et al. used glow discharge plasma treatment instead of thermal calcination to increase the oxygen vacancy concentration, and thus, enhanced nickel dispersion [23]. In addition to the optimization of the synthesis method, the introduction of defects over redox support through the reductive gas (such as CO and H_2_) treatment under a high temperature or pressure conditions has been a feasible strategy [24,25]. Therefore, treatment of the Ni–CeO_2_ by direct hydrogen without high-temperature air calcination may be a feasible way to increase the oxygen vacancy concentration of catalyst.

In this study, a CeO_2_-supported, Ni-based catalyst was prepared through direct reduction of dried catalyst precursors without calcination and applied for hydrogen production from glycerol steam reforming for the first time. As a comparison, another Ni–CeO_2_ catalyst was synthesized with thermal calcination followed by H_2_ reduction. The physicochemical properties of catalysts were characterized by various techniques. The influences of the pretreatment conditions on the redox property, metal-support interaction, crystallite size, reducibility and dispersion of metal, and surface oxygen vacancies have been systematically explored. The coke deposition on the spent catalysts was also analyzed, and the deactivation mechanism of catalysts was revealed.

## 2. Materials and Methods

### 2.1. Materials and Catalyst Preparation

Cerium nitrate hexahydrate (Ce(NO_3_)_3_·6H_2_O), ethylene glycol, acetic acid, and nickel nitrate (Ni(NO_3_)_2_·6H_2_O) were supplied by Sinopharm (Shanghai, China).

CeO_2_ spheres were first prepared by the hydrothermal method to be used as the catalyst support. Typically, an aqueous solution of Ce(NO_3_)_3_·6H_2_O (0.08 M) was added to the mixed solutions of 2 mL acetic acid and 52 mL ethylene glycol solution under a stirring state. The above mixture was stirred vigorously for 30 min to obtain a milky white mixture. Then, the milky, white mixture was transferred into a Teflon vessel inside a stainless steel autoclave to perform a hydrothermal treatment at 180 °C for 200 min to acquire the CeO_2_ sphere.

**Figure 1:**
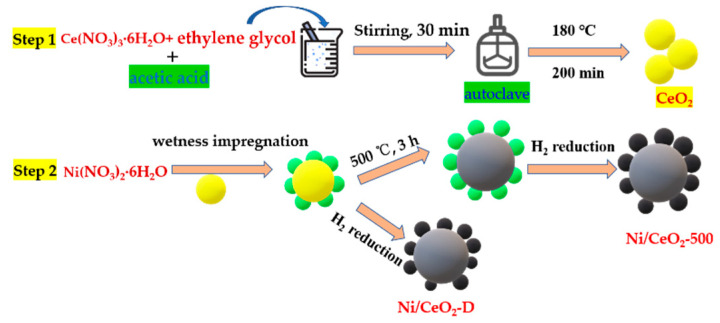
Schematic illustration of the formation evolution of Ni/CeO_2_-500 and Ni/CeO_2_-D.

CeO_2_-supported, Ni-based catalysts were prepared via a wetness impregnation method using Ni(NO_3_)_2_·6H_2_O as nickel precursors. The nominal nickel loading was 7.5 wt%. First, a certain amount of Ni(NO_3_)_2_·6H_2_O was dissolved in deionized water, then CeO_2_ sphere was added, followed by stirring for 30 min. The obtained sample was divided equally into two parts. A portion of the samples was dried at 100 °C for 12 h and then calcinated at 500 °C for 3 h (labeled as Ni/CeO_2_-500). The other part was only dried at 100 °C for 12 h without calcination (named as Ni/CeO_2_-D). The schematic illustration of the catalyst preparation process is shown in Figure 1.

### 2.2. Catalyst Characterization

N_2_ adsorption–desorption was performed on a Quantachrome NOVA 4200e (New York, NY, USA) to measure the physical characteristics of the synthesized Ni–CeO_2_ catalysts. All samples to be tested were pretreated at 300 °C for 3 h, and the nitrogen adsorption and desorption process was then carried out under liquid nitrogen temperature conditions (−196 °C). Brumauer–Emmett–Teller (BET) and Barrett–Joyner–Halanda (BJH) models were used to calculate the specific surface area and pore size distribution, respectively. The pore volume was calculated by the amount of N_2_ adsorbed at P/P_0_ = 0.9908. X-ray diffractograms (XRD) were measured by a Rigaku D/max-1200 diffractometer (Tokyo, Japan) equipped with Cu Kα radiation (λ= 1.5406 Å), and the scanning range was set from 10° to 80°. The crystalline size of the related metal species was calculated using the Scherer equation by Jade 6.0 software. X-ray photoelectron spectroscopy (XPS) was executed on an ESCALAB 250Xi spectrometer (Waltham, MA, USA) with a monochromatic Al Kα (1486.6 eV) radiation source. The binding energies were calibrated on the base of the hydrocarbon C1s peak at 284.8 eV. Transmission electron microscope (TEM) and HRTEM photographs were performed on FEI Tecnai G2 F20 and FEI Talos F200S (Waltham, MA, USA). Raman spectra were performed through a micro-Raman system (Dilor Labram Model, Thermo Scientific Inc., Waltham, MA, USA) and the measurement wavelength was set to 532 nm. The amount of the carbon deposition of the spent catalysts was characterized by the Thermogravimetric Analyzer (TGA 4000, PerkinElmer, Waltham, MA, USA). The operating temperature was from 30 to 800 °C, under air atmosphere. The inductively coupled plasma–optical emission spectroscopy (ICP–OES) (Prodigy, Leeman Labs, Hudson, NH, USA) was used to examine the elemental contents of the prepared catalysts.

The oxygen storage capacity (OSC) experiment was performed on a TCD detector to explore the oxygen storage capacity of catalysts. A 50 mg sample was first reduced in situ at 500 °C, under a 50% H_2_/N_2_ flow for 1 h, and then He (30 mL min^−1^) was purged for 10 min to remove the residual hydrogen. The catalysts, after reduction, were pretreated, in-situ, at 500 °C under 10% O_2_/He flow (30 mL min^−1^) for 30 min and then He (30 mL min^−1^) was purged for 30 min. They were pulsed with 50 mol of 100% CO and He as the carrier stream to reduce ceria completely. The H_2_ temperature-programed reduction (H_2_-TPR) experiment was used to explore the differences in redox behavior of catalysts. Briefly, the samples to be tested were first treated at 400 °C for 30 min under a mixed gas of 5 vol% of O_2_/Ar. The sample was then cooled to100 °C and the mixed gas flow of 10% H_2_/Ar was introduced. In the test stage, the samples were heated to 800 °C with a rate of 10 °C/min and the TCD signal was recorded continuously. The H_2_ pulse experiment was used to determine the exposed surface area and the dispersion of active nickel, and the detailed steps were shown in our previous articles [26]. Hydrogen chemisorption was pulsed by 10% H_2_/Ar at 40 °C, and the samples were reduced at 500 °C for 1 h prior to chemisorption. The calculation of the exposed surface area and the dispersion of active nickel followed the method of the previous article [26]. Assuming that hydrogen was adsorbed on the surface of nickel atoms in an atomic state, one nickel atom adsorbed one hydrogen atom and the surface area of one atom was 6.49 × 10^−18^ m^2^/atom.

The turnover rate (TOF) was applied to compare the molar amount of converted glycerol per unit time at a single active site in different catalysts, and the relevant calculation formulas were as follows:

(1) $$\mathrm{TOF}=\frac{N}{\mathrm{tM}}$$

(2) $$N=t\cdot v\cdot X_{\mathrm{Glycerol}}$$

(3) $$M=m_{\mathrm{Ni}}\cdot D/59$$

The symbols are as follows: X_Glycerol_, conversion of glycerol; m_Ni_, nickel content; M, the number of surface nickel atoms; N, the molar amount of glycerol converted during the reaction time; t, reaction time; v, Glycerol flow rate; and D, nickel dispersion.

### 2.3. Catalytic Test

Steam reforming of Glycerol (GSR) for hydrogen production was conducted in a fixed-bed reactor under atmospheric pressure with a gas hourly space velocity (GHSV) of 28,500 h^−1^. 

Typically, the catalyst (200 mg, 40–60 mesh) was reduced by 50% H_2_/N_2_ (60 mL/min) at 500 °C. The mixed liquid of H_2_O and C_3_H_8_O_3_ (H_2_O: C_3_H_8_O_3_ = 9:1, 0.02 mL/min) was injected by an HPLC pump, while the inert N_2_ (60 mL/min) was added to the vaporizer at an evaporation temperature of 220 °C. The reaction temperature of the catalytic activity test was 400~600 °C and the reaction interval temperature was 50 °C. The relative experimental data were obtained under steady-state conditions. Furthermore, the catalytic stability test of the catalyst was conducted at 500 °C. The conversion of glycerol into gaseous products, the hydrogen yield, and the gaseous product selectivity were calculated with the following equations. 

The conversion of glycerol into gaseous products

(4) $$X_{\mathrm{Gas}}=\frac{\begin{array}{cccccc} \mathrm{moles} & \mathrm{of} & C & \mathrm{in} & \mathrm{gas} & \mathrm{product} \end{array}}{\begin{array}{ccccccc} 3\times & \mathrm{moles} & \mathrm{of} & \mathrm{glycerol} & \mathrm{in} & \mathrm{feed} & \end{array}}\times 100\%$$

The yield of H_2_ was calculated by

(5) $$Y_{H_{2}}=\frac{\begin{array}{cc} \begin{array}{cc} \begin{array}{cc} \mathrm{moles} & \mathrm{of} \end{array} & H_{2} \end{array} & \begin{array}{cc} \mathrm{in} & \mathrm{gas}\begin{array}{c} \mathrm{product} \end{array} \end{array} \end{array}}{\begin{array}{ccc} \begin{array}{ccc} \begin{array}{cc} \mathrm{moles} & \mathrm{of} \end{array} & \mathrm{glycerol} & \mathrm{in} \end{array} & \mathrm{feed} & \end{array}}$$

The selectivity of H_2_ was calculated by

(6) $$S_{H_{2}}=\frac{\begin{array}{cc} \begin{array}{cc} \begin{array}{cc} \mathrm{moles} & \mathrm{of} \end{array} & H_{2} \end{array} & \mathrm{produced} \end{array}}{\begin{array}{ccc} \begin{array}{ccc} \begin{array}{cc} \mathrm{moles} & \mathrm{of} \end{array} & C & \mathrm{in} \end{array} & \mathrm{gas} & \mathrm{products} \end{array}}\times \frac{3}{7}\times 100\%$$

The selectivity of CO, CO_2_, and CH_4_ were calculated by

(7) $$S_{i}=\frac{\begin{array}{cccc} & \mathrm{moles} & \mathrm{of} & i \end{array}}{\begin{array}{ccc} \begin{array}{ccc} \begin{array}{cc} \mathrm{moles} & \mathrm{of} \end{array} & C & \mathrm{in} \end{array} & \mathrm{gas} & \mathrm{production} \end{array}}\times 100\%$$

Here, C(i) is the concentration of each carbonaceous substance.

## 3. Results and Discussion

### 3.1. Structure and Properties of Calcined/Reduced Catalysts

**Figure 2:**
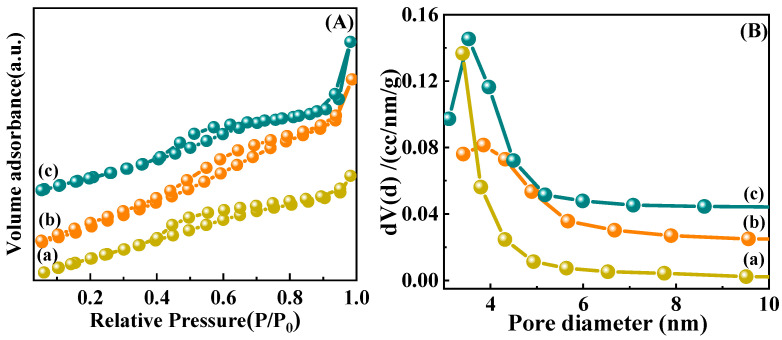
N_2_ adsorption–desorption isotherm plots (**A**) and pore distributions (**B**) of pure CeO_2_ (**a**), Ni/CeO_2_-500 (**b**), and Ni/CeO_2_-D (**c**).

N_2_ adsorption–desorption characterization is applied to study the textural properties of pure CeO_2_ support, Ni/CeO_2_-500, and Ni/CeO_2_-D samples. The adsorption–desorption isotherms, as well as the corresponding pore size distribution chart, are displayed in Figure 2A,B, respectively. It can be seen from Figure 2A that the adsorption isotherms and hysteresis loops of the samples were categorized as type IV and H2-type, indicating the presence of mesopores [27,28]. After being loaded by Ni, the specific surface area of the catalyst decreased, and the pore size became slightly larger. The pore size of Ni/CeO_2_-500 was in the concentration range of 2.5~7.5 nm, with the average pore size of 4.3 nm (Figure 2B). The pore size of Ni/CeO_2_-D was in the range of 2.5~5.6 nm, with the average pore size of 4.1 nm. Besides, the slightly smaller specific surface area and pore volume of Ni/CeO_2_-500 compared to Ni/CeO_2_-D (Table 1) suggested that the thermal calcination treatment had a negative impact on the structural properties of the sample.

**Figure 3:**
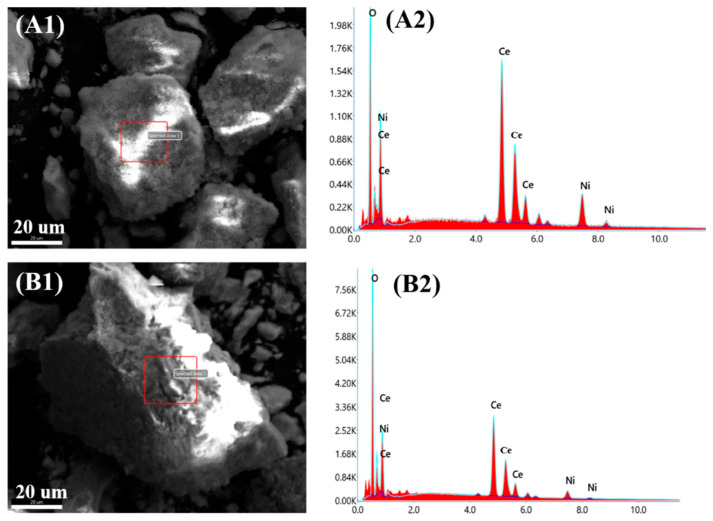
EDS mapping and the elemental distribution of the reduced Ni/CeO_2_-500 (**A1**,**A2**) and Ni/CeO_2_-D (**B1**,**B2**).

To verify the elemental composition of the catalysts, the EDS analysis was applied, and the results are shown in Figure 3. The characterization results demonstrated that only Ni, Ce, and O elements presented in both the Ni/CeO_2_-500 (Figure 3(A1,A2)) and Ni/CeO_2_-D (Figure 3(B1,B2)) catalysts, and no other impurity elements were observed. In addition, the inductively coupled plasma–optical emission spectroscopy (ICP–OES) was applied to further quantify the amount of nickel doping in the catalyst, and the results are shown in Table 1. 

**Figure 4:**
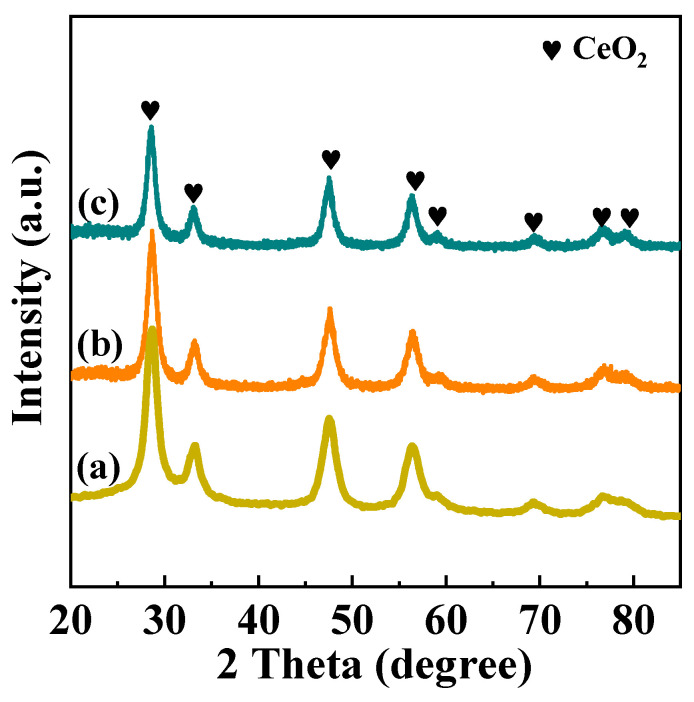
XRD patterns of the calcined CeO_2_ (**a**), Ni/CeO_2_-500 (**b**), and Ni/CeO_2_-D (**c**).

XRD patterns of pure CeO_2_, Ni/CeO_2_-500, and Ni/CeO_2_-D are shown in Figure 4. From the XRD standard pattern card (JCPDS PDF # 43-1002), it was determined that both catalysts had a typical cubic fluorite structure. In Figure 3, both catalysts obtained the diffraction peaks at (2θ = 27.9°, 32.9°, 47.1°, 56.1°, 58.9°, 68.9°, 76.8°, and 79.2°), which were attributed to (111), (200), (220), (311), (222), (400), (331), and (420) planes of CeO_2_, respectively. In comparison with Ni/CeO_2_-500, the diffraction pattern of Ni/CeO_2_-D was slightly wider and weaker, which implied that the grain size of Ni/CeO_2_-D was smaller. In addition, no diffraction peaks attributed to nickel species were observed on the catalysts, indicating that the nickel species supported on the CeO_2_ was highly dispersed, or the nickel nanoparticles were rather small.

The lattice parameters of CeO_2_ for pure CeO_2_, Ni/CeO_2_-500, and Ni/CeO_2_-D are calculated by the Bragg equation, and the result is listed in Table 1. The lattice parameters of pure CeO_2_ were 5.412 Å, and the lattice parameters of Ni/CeO_2_-500 and Ni/CeO_2_-D decreased to 5.406 and 5.409 Å after Ni doping, respectively. It inferred that a certain amount of nickel ions were incorporated into ceria to substitute Ce^4+^ ion and then shrunk the unit cell parameter of ceria due to the ion radius of Ni^2+^ (0.069 nm), which was smaller than that of the Ce^4+^ ion (0.096 nm) [29,30]. In addition, a greater lattice contraction of Ni/CeO_2_-500 implied that a larger portion of nickel ions replaced the Ce^4+^ ion in Ni/CeO_2_-500 than that of Ni/CeO_2_-D, which suggested that a stronger interaction between Ni and CeO_2_ of Ni/CeO_2_-500 was generated. 

**Figure 5:**
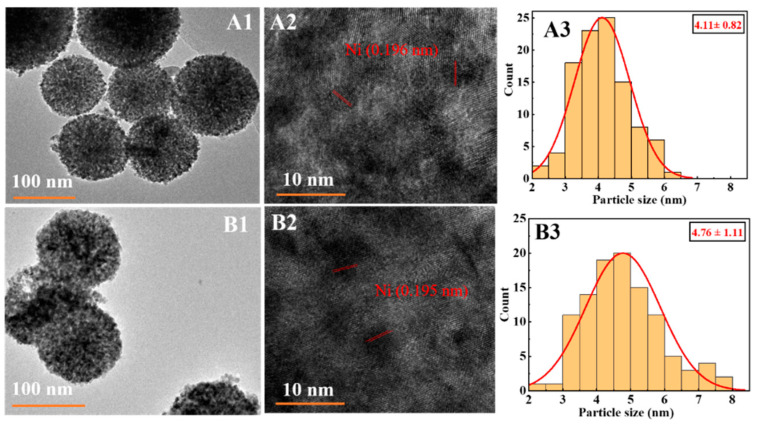
TEM (**A1**), HRTEM images (**A2**), and associated Ni particle size distribution histograms (**A3**) for reduced Ni/CeO_2_-D. TEM (**B1**), HRTEM images (**B2**), and associated Ni particle size distribution histograms (**B3**) for reduced Ni/CeO_2_-500.

To further investigate the dispersion of nickel, the TEM mapping analysis of the catalyst was performed, and the result is shown in Figure 5. CeO_2_ sphere-supportednickel-based catalysts were successfully synthesized with nickel dispersed on the surface of CeO_2_ spheres (Figure 5A1,A2,B1,B2). Compared to the pretreatment means of thermal calcination followed by reduction, the results clearly showed that the direct hydrogen reduction facilitated the acquisition of uniformly dispersed, smaller nickel nanoparticles (Figure 5A3, d = 4.11 ± 0.82 nm). Besides, the average size of nickel nanoparticles for both Ni/CeO_2_-500 and Ni/CeO_2_-D was less than 5 nm, which might lead to no characteristic diffraction peaks of nickel on XRD. In addition, the particle size distribution of nickel species showed that direct hydrogen reduction treatment instead of thermal calcination followed by reduction promoted a 13.7% decrease of nickel particle size under the same nickel loading conditions. The related results demonstrated that the direct reduction treatment was beneficial to obtain uniform small nickel nanoparticles.

**Figure 6:**
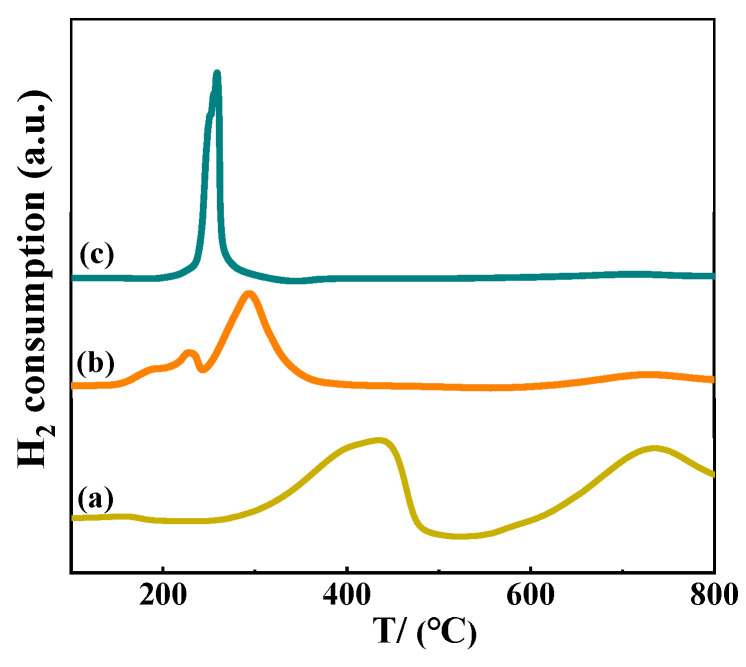
H_2_–TPR patterns of the pure CeO_2_ (**a**), Ni/CeO_2_-500 (**b**), and Ni/CeO_2_-D (**c**).

H_2_-TPR characterization of pure CeO_2_, Ni/CeO_2_-500, and Ni/CeO_2_-D was executed to explore the reduction characteristics of Ni species (Figure 6). The hydrogen consumption peaks of the bare ceria occurred in the range of 300–500 °C and above 650 °C, and were assigned to the reduction of surface oxygen and bulk oxygen, respectively [31]. The weak peak above 600 °C in Ni/CeO_2_-500 and Ni/CeO_2_-D was attributed to the reduction of bulk oxygen according to the H_2_–TPR profiles of pure CeO_2_ support. For Ni/CeO_2_-500, two small hydrogen consumption peaks in the 150 to 240 °C range might be due to the reduction of adsorbed oxygen species on oxygen vacancies, and the major reduction peak around 300 °C was assigned to the reduction of NiO [23,32]. Because nickel nitrate could be decomposed when exposed to high temperatures, the main reduction peak, concentrated on 180 to 340 °C of Ni/CeO_2_-D, probably belonged to the decomposition of nickel nitrate and the reduction of nickel species. Adsorbed oxygen species could also not be excluded [28]. The reduction temperature of NiO in Ni/CeO_2_-D was lower than that of Ni/CeO_2_-500, indicating that nickel species on Ni/CeO_2_-D were more easily reduced than on Ni/CeO_2_-500, and the weaker interaction between Ni species and CeO_2_ of Ni/CeO_2_-D might be acquired.

**Figure 7:**
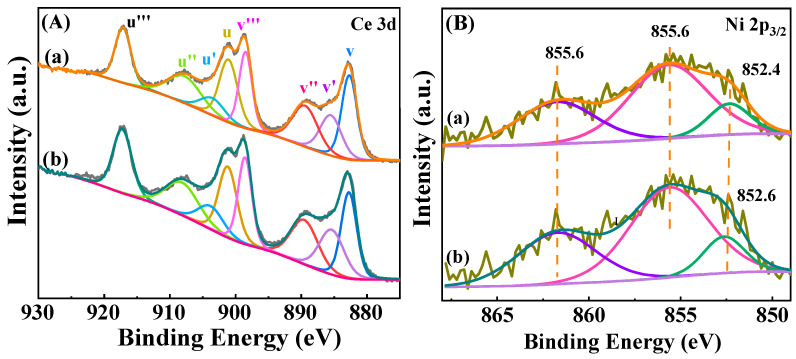
XPS spectra of Ce3d (**A**) and Ni 2d (**B**) of reduced Ni/CeO_2_-500 (**a**) and Ni/CeO_2_-D (**b**).

**Figure 8:**
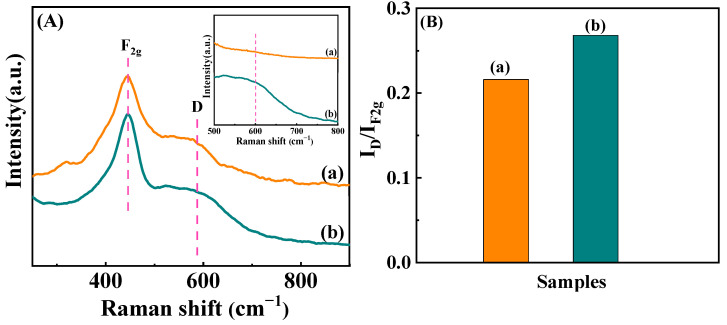
Raman spectra (insert: the expended spectra between 500–800 cm^−1^) (**A**) and the ratios of I_D_/I_2g_ (**B**). Ni/CeO_2_-500 (**a**) and Ni/CeO_2_-D (**b**).

To investigate the chemical state of the elements on the catalyst, Ce 3d XPS spectra of the reduced Ni/CeO_2_-500 and Ni/CeO_2_-D were analyzed (Figure 7A). The Ce 3d XPS spectra were fitted to eight peaks, i.e., four pairs: v/u, v′/u′, v″/u″, and v‴/u‴ by Casa XPS [33]. The characteristic peaks of u′ and v′ peaks are assigned to the Ce^3+^ species, while peaks denoted as u/v, u″/v″, and u‴/v‴ are classified as Ce^4+^ species [34]. The percentage of peak area attributed to the Ce^3+^ of Ni/CeO_2_-D was 18.81%, and obtained from the split-peak fitting, which was higher than that of Ni/CeO_2_-500 (18.05%). Considering the fact that Ce^3+^ was transformed from Ce^4+^ and the transformation process was accompanied by the generation of oxygen vacancies, higher oxygen vacancy concentrations were considered to be present in Ni/CeO_2_-D. This result was in good agreement with the following Raman results (Figure 8).

Ni 2p_3/2_ spectra are shown in Figure 5B. Peaks attributed to metallic Ni at 852.6 eV and 852.4 eV were observed on Ni/CeO_2_-500 and Ni/CeO_2_-D, respectively [35]. Additionally, a high-intensity peak around 855.6 eV and a low-intensity and broad peak around 861.6 eV were detected for both reduced catalysts. The former one belonged to the Ni 2p_3/2_ of NiO, and the other one was attributed to its satellite peak [36]. The presence of NiO stemmed from the fact that part of the NiO was oxidized by exposure to air. The elemental percentages of different chemical states of nickel elements were calculated and are shown in Table 2. It revealed that the ratio of Ni^0^/(Ni^0^ + Ni^2+^) of Ni/CeO_2_-D (25.01%) was higher than that of Ni/CeO_2_-500 (24.57%), which indicated that Ni/CeO_2_-D obtained a higher reduction degree than Ni/CeO_2_-500. At the same time, the ratio of Ce^3+^/(Ce^3+^ + Ce^4+^) on Ni/CeO_2_-D (18.81%) was greater than on Ni/CeO_2_-500 (18.05%) through the integrated peak area calculation (Table 2), which meant that Ni/CeO_2_-D achieved higher concentrations of oxygen vacancies. Combing the calculation results of the Ce^3+^ and Ni^0^ ratios of the reduced Ni/CeO_2_-500 and Ni/CeO_2_-D, it suggested that direct reduction treatment facilitated the generation of more oxygen vacancies and higher nickel reduction.

Raman spectroscopy was utilized to further investigate the intensity of lattice defects on Ni/CeO_2_-500 and Ni/CeO_2_-D, and the result is shown in Figure 8. The strong band around 455 cm^−1^ (F_2g_) was classified as the stretching vibration of the cerium dioxide fluorite structure. The other weak D band (∼590 cm^−1^) was attributed to the defects-induced vibration mode, and it was the stretching vibration of the oxygen vacancy due to the presence of Ce^3+^ in our catalytic system [37]. The integrated peak area ratio of F_2g_ and D (I_D_/I_F2g_) is commonly used to quantify the relative content of oxygen vacancies. As presented in Figure 8B, the ratio of I_D_/I_F2g_ over Ni/CeO_2_-D (I_D/_ I_F2g_ = 0.268) was larger than that of Ni/CeO_2_-500 (I_D_/I_F2g_ = 0.216), demonstrating that more oxygen vacancies were generated in Ni/CeO_2_-D. In addition, the results helped to understand the better nickel dispersion of Ni/CeO_2_-D: surface defects could act as anchoring sites to stabilize the nickel nanoparticles and inhibit their aggregation, which led to a higher nickel dispersion. 

### 3.2. Activity Evaluation of Ni/CeO_2_-500 and Ni/CeO_2_-D Catalysts

**Figure 9:**
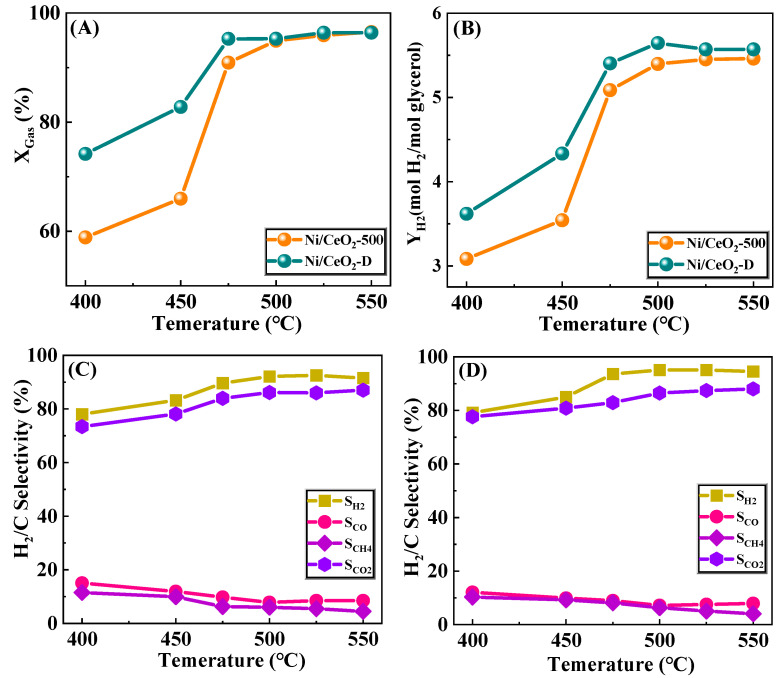
Catalytic activity of Ni/CeO_2_-500 and Ni/CeO_2_-D. Glycerol conversion (**A**), H_2_ yield (**B**), selectivity of H_2_, CO, CH_4_, and CO_2_ of Ni/CeO_2_-500 (**C**), and Ni/CeO_2_-D (**D**).

The evaluation of glycerol conversion, H_2_ yield, and the selectivity of gaseous products of Ni/CeO_2_-D and Ni/CeO_2_-500 with the increase of reaction temperature has been discussed (Figure 9). The glycerol conversion and H_2_ yields of both Ni/CeO_2_-D and Ni/CeO_2_-500 improved significantly with the rise of the reaction temperature, indicating that the high temperature facilitated the catalytic reaction, which was consistent with the GSR being an endothermic reaction (Figure 9A,B) [3]. Moreover, the conversion of glycerol, as well as the H_2_ yield of Ni/CeO_2_-D, were greater than those of Ni/CeO_2_-500 over the total reaction temperature interval, especially in the low-temperature reaction region (400–475 °C). When obtaining the same conversion and hydrogen (95%) and H_2_ yield (5.4 mol/mol glycerol), the catalytic temperature of Ni/CeO_2_-D was 475 °C (T_95_ = 475 °C), while the reaction temperature of Ni/CeO_2_-500 was 500 °C (T_95_ = 500 °C), indicating that the pretreatment method of direct reduction, without the calcination step, could effectively improve the low-temperature catalytic activity. 

The selectivity of gaseous products for Ni/CeO_2_-D and Ni/CeO_2_-500 is shown in Figure 9C,D. For both Ni/CeO_2_-D and Ni/CeO_2_-500, the selectivity of CH_4_ decreased with rising reaction temperature, demonstrating that the methanation process was inhibited by high temperatures, which was consistent with the methanation reaction being exothermic. The selectivity of CO of both Ni/CeO_2_-D and Ni/CeO_2_-500 showed a trend of the first drop and then the rise, and they reached the minimum at 475 °C and 500 °C, respectively. In addition, the selectivity of CO of Ni/CeO_2_-D was higher than that of Ni/CeO_2_-500, which were all in the whole-reaction temperature range. Combining the high selectivity of H_2_ and low CO selectivity of Ni/CeO_2_-D, it was presumed that the water–gas shift reaction was well promoted in Ni/CeO_2_-D. Turnover frequency (TOF) of Ni/CeO_2_-D and Ni/CeO_2_-500 was used to further calculate the conversion of glycerol per unit of the nickel-active site, and the result is shown in Figure 9 and Table 3. The TOF value of Ni/CeO_2_-D (7.63 s^−1^) was higher than that of Ni/CeO_2_-500 (7.45 s^−1^), further proving the high catalytic activity of Ni/CeO_2_-D for GSR. 

**Figure 10:**
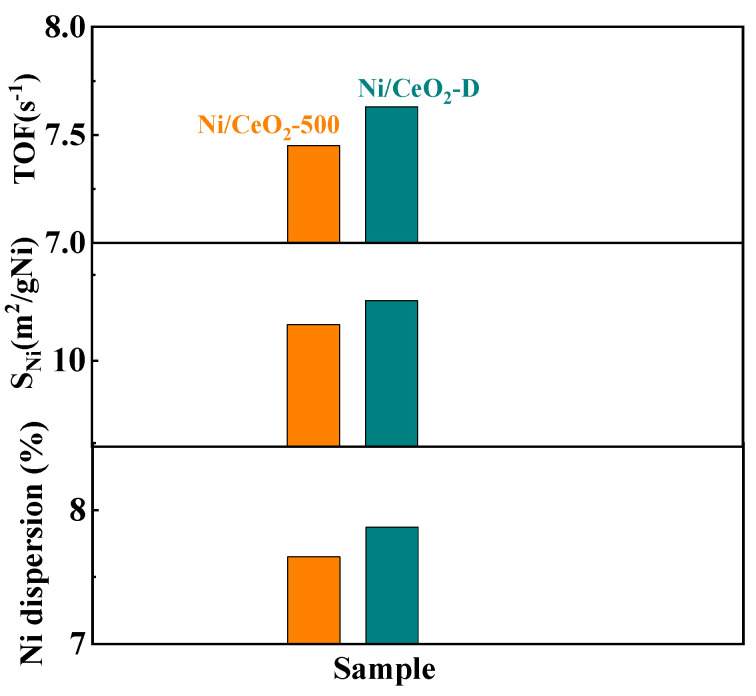
The dispersion and exposed surface areas of active nickel and TOF value.

In GSR, Ni^0^ as the most important active site can efficiently break C–H and C–C bonds of glycerol and facilitate the water–gas shift reaction [1,38]. To quantify the number of active sites, hydrogen pulse means were used to measure the adsorbed hydrogen values, and thus, calculated the exposed surface area of active Ni^0^ for both Ni/CeO_2_-D and Ni/CeO_2_-500 catalysts (Figure 10 and Table 3). The Ni^0^ dispersion of Ni/CeO_2_-D (7.87%) was higher than that of Ni/CeO_2_-500 (7.65%). Therefore, Ni/CeO_2_-D obtained a greater exposed surface area of active Ni^0^ (10.35 m^2^/gNi) than that of the Ni/CeO_2_-500 (10.21 m^2^/gNi), which benefited from high nickel dispersion and reduction. In addition, due to the high surface area of active Ni^0^ and TOF value, Ni/CeO_2_-D obtained a high glycerol conversion and H_2_ yield. Furthermore, oxygen vacancies are thought to promote the activation of water to generate more OH groups, to facilitate the production of hydrogen through steam reforming [39,40]. In the steam reforming of glycerol, the water–gas shift reaction ($\mathrm{CO}\;+{\;H}_{2}O\to {\mathrm{CO}}_{2}+H_{2}$) is one of the most important process reactions, and the dissociative chemisorption of H_2_O on the catalyst surface is an important reaction step affecting the rate of hydrogen production [3,16]. Therefore, it is speculated that oxygen vacancies also played a crucial role in the glycerol-steam-reforming reaction. By comparing the catalytic activities of Ni/CeO_2_-500 and Ni/CeO_2_-D with different oxygen vacancy concentrations, it was also confirmed that Ni/CeO_2_-D with higher concentrations of oxygen vacancies obtained better catalytic performance.

### 3.3. Catalytic Stability Test and Characterization of the Spent Catalyst

#### 3.3.1. Catalytic Stability Test

**Figure 11:**
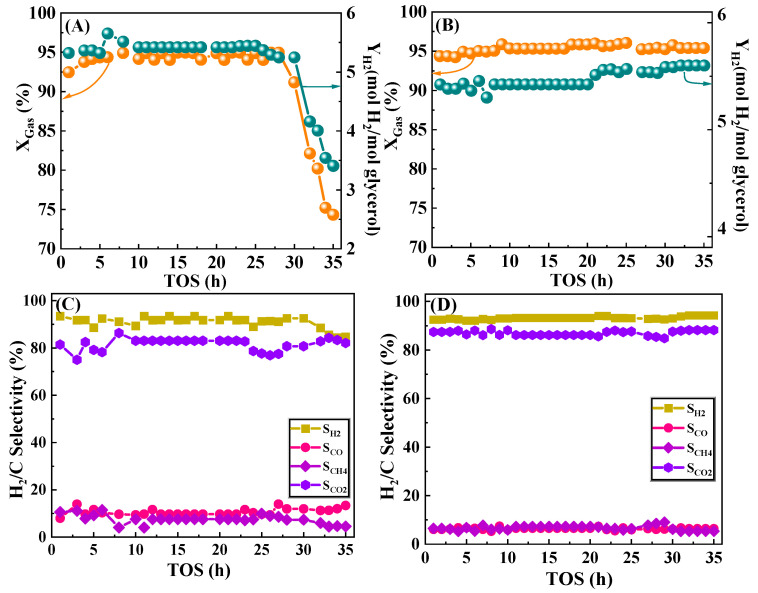
Stability test of Ni/CeO_2_-500 and Ni/CeO_2_-D. Glycerol conversion and H_2_ yield of Ni/CeO_2_-500 (**A**) and Ni/CeO_2_-D (**B**). Selectivity of H_2_, CO, CH_4_, and CO_2_ of Ni/CeO_2_-500 (**C**) and Ni/CeO_2_-D (**D**). Reaction conditions: nC_3_H_8_O_3_: nH_2_O = 1:9, GHSV = 28500 mL h^−1^ gcat^−1^, reaction temperature = 500 °C (Ni/CeO_2_-500) /475 °C (Ni/CeO_2_-D), and atmospheric pressure.

The evolution of glycerol conversion, H_2_ yield, and the selectivity of the products over time for Ni/CeO_2_-500 and Ni/CeO_2_-D was investigated, and the results are presented in Figure 11. To compare the catalytic stability of the two catalysts under the same initial glycerol conversion condition, 500 °C was chosen as the temperature for the stability experiment of Ni/CeO_2_-500, and 475 °C as the temperature for the stability experiment of Ni/CeO_2_-D, based on the activity results in Figure 8. Ni/CeO_2_-500 (Figure 11A) was almost inactivated during the first 25 h, but the glycerol conversion dropped persistently during the next 10 h of time, and it was below 75% after 35 h of reaction. The deactivation revealed the rapid loss of active sites on Ni/CeO_2_-500 for glycerol degradation. Ni/CeO_2_-D (Figure 11B) still maintained a high glycerol conversion and hydrogen yield throughout the 35 h stability experiments without deactivation, suggesting that the active sites have consistently maintained high catalytic activity. In addition, the present work was compared with previous work and the results are presented in Table 4.

The effect of reaction time on the selectivity of H_2_ and carbonaceous gaseous products (CO, CH_4_, and CO_2_) was also investigated (Figure 11C,D). The relevant data were obtained under steady-state conditions. The selectivity of H_2_, CO, CH_4_, and CO_2_ for the Ni/CeO_2_-D catalyst was maintained well at 94%, 6%, 7%, and 87%, respectively, throughout the reaction, without major fluctuations. However, the H_2_ selectivity decreased substantially from 91.3 to 84.5% for Ni/CeO_2_-500, and the CO selectivity increased from 9.7 to 11.2%. It estimated that the water–gas shift reaction over Ni/CeO_2_-500 was inhibited, thus the selectivity of CO selectivity rose and resulted in a decrease in the H_2_ selectivity. In addition, CO disproportionation is one of the important triggers for carbon deposition in nickel-based catalysts, and high CO selectivity may exacerbate the rate of carbon deposition. The high carbon generation rate hastened the encapsulation of the Ni^0^ active center, which further aggravated the deactivation of the catalyst, and thus, Ni/CeO_2_-500 exhibited poor stability. To investigate the causes for the differences in stability between Ni/CeO_2_-500 and Ni/CeO_2_-D, XRD, TG, Raman, and TEM were applied to characterize the spent catalysts.

#### 3.3.2. Structure and Properties of Spent Catalysts

The spent Ni/CeO_2_-500 and Ni/CeO_2_-D after 35 h (named as Ni/CeO_2_-500-35 and Ni/CeO_2_-D-35, respectively) of catalytic stability experiments were characterized through XRD, TG/TDA, Raman, and TEM to probe the causes of different catalytic stability performance.

**Figure 12:**
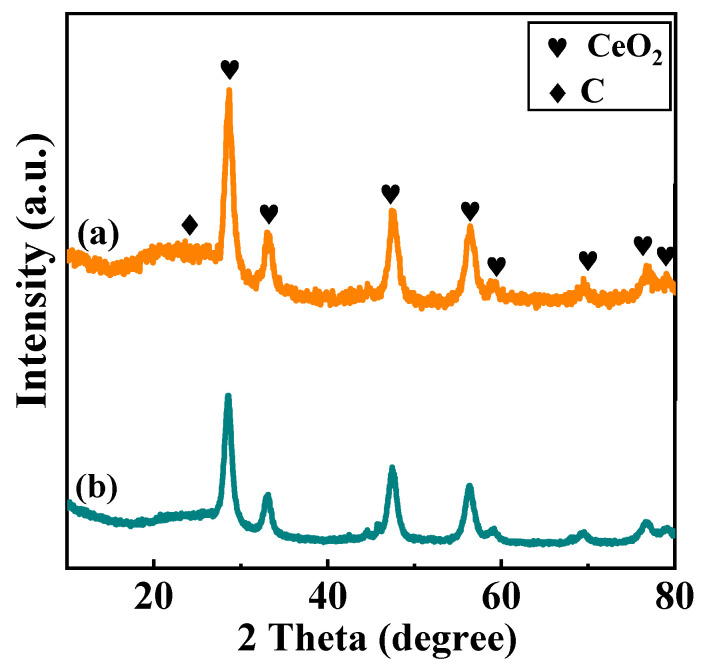
XRD patterns of the spent Ni/CeO_2_-500 (**a**) and Ni/CeO_2_-D (**b**).

WXRD patterns of spent Ni/CeO_2_-500-35 and Ni/CeO_2_-D-35 are shown in Figure 12. For both Ni/CeO_2_-500-35 and Ni/CeO_2_-D-35, only the diffraction peaks that belonged to the CeO_2_ phase were observed, and no characteristic peaks assigned to NiO or Ni were detected. This suggested that no obvious aggregation or growth of nickel nanoparticles in the catalyst occurred, and no oxidation of monolithic nickel happened. In addition to the diffraction peak belonging to CeO_2_ being detected, a new peak around 2θ = 26.5° assigned to the graphitic carbon peak (JCPDS Card No. 41-1487) was observed on Ni/CeO_2_-500-35, suggesting a certain amount of graphite carbon was deposited on Ni/CeO_2_-500-35. In contrast, the diffraction peaks of graphitic carbon for Ni/CeO_2_-D-35 were weaker, indicating less graphitic carbon deposition on the surface of Ni/CeO_2_-D-35.

**Figure 13:**
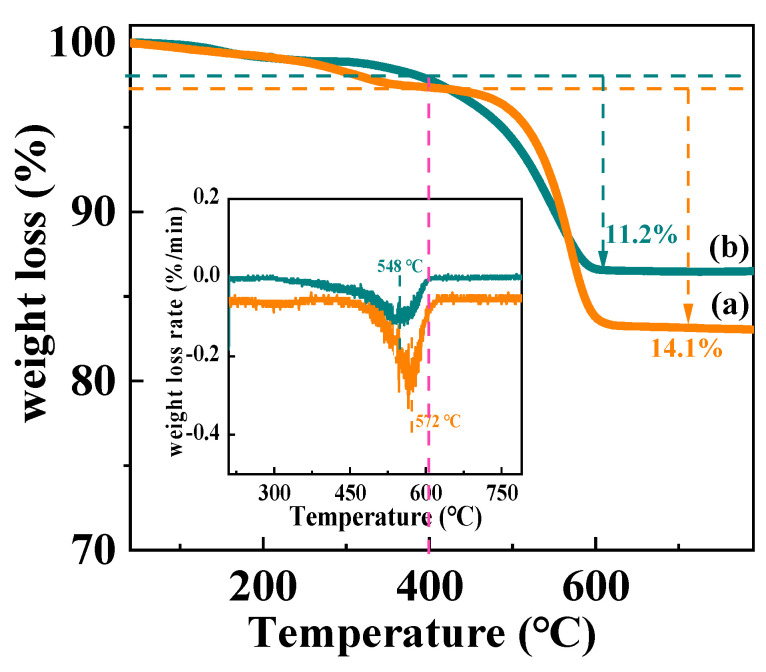
TGA curves of spent Ni/CeO_2_-500 (**a**) and Ni/CeO_2_-D (**b**).

The amount and nature of carbon deposition on the catalyst were characterized by TG via mass reduction induced by the consumption of the carbon deposition under an airy atmosphere (Figure 13). According to previous research, the small initial mass reduction below 200 °C was ascribed to the volatilization of moisture and volatiles from the catalyst [47]. The second weightless temperature zone between 200–400 °C was caused by the oxidation of easily removable, physically adsorbed carbon species [48]. The major weightless temperature region was attributed to the oxidation of bulky carbonaceous species or hard carbon on the Ni/CeO_2_-500-35 and Ni/CeO_2_-D-35 [27]. The weight loss percentages ascribed to the hard carbon of Ni/CeO_2_-500-35 and Ni/CeO_2_-D-35 were 14.1% and 11.2%, respectively, indicating the hard carbon deposition on Ni/CeO_2_-D-35 was higher than that on Ni/CeO_2_-500-35. In addition, the quantity of carbon deposition (mol carbon/mol glycerol) and the velocity of carbon accumulation of Ni/CeO_2_-500-35 was higher than that of Ni/CeO_2_-D-35 (Table 5). Furthermore, the DTG profiles (insert) were studied to investigate the weight loss temperature interval and maximum weight loss temperature of Ni/CeO_2_-500-35 and Ni/CeO_2_-D-35. The weightless temperature region of Ni/CeO_2_-D-35 was around 400–605 °C, with the major weight loss rate at 548 °C, and the weightless temperature region of Ni/CeO_2_-500-35 was around 400–635 °C, with the major weight loss rate at 572 °C. The higher major weight removal temperature of Ni/CeO_2_-500-35 than that of Ni/CeO_2_-D-35 suggested that the carbon deposition on Ni/CeO_2_-500-35 was more difficult to be removed.

**Figure 14:**
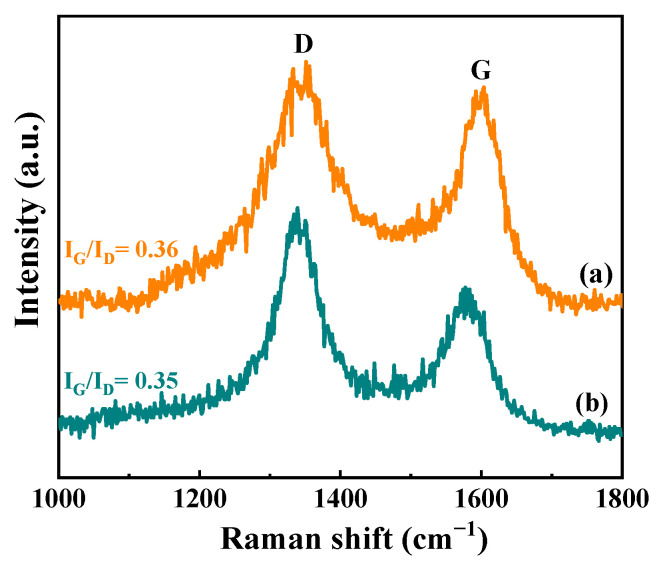
Raman spectra of spent Ni/CeO_2_-500 (**a**) and Ni/CeO_2_-D (**b**).

Raman spectroscopy was employed to further study the nature of carbon deposition on Ni/CeO_2_-500-35 and Ni/CeO_2_-D-35 (Figure 14). Two major peaks around 1345 and 1595 cm^−1^ were observed in both the catalysts, which were classified as D and G bands of carbon deposition, respectively. D band is often thought of as the vibration of carbon atoms in disordered aromatic structures [49]. The G band is usually considered to be the characteristic band of condensed, ordered, or graphitic aromatic structures [50]. In addition, the band intensity ratio (I_G_/I_D_) is normally used to evaluate the degree of graphitization of carbon deposition on the spent catalysts [51]. The I_G_/I_D_ ratio values of Ni/CeO_2_-500-35 and Ni/CeO_2_-D-35 are 0.36 and 0.35, respectively. As for the same reaction time, a higher graphitic degree of carbon deposition on Ni/CeO_2_-500-35 was obtained than that on Ni/CeO_2_-D-35.

In GSR, methane cracking and the disproportionation reaction of carbon monoxide are the two most serious side reactions that lead to carbon deposition on the spent catalysts. The high selectivity of CO and CH_4_ of Ni/CeO_2_-500-35 (Figure 11) resulted in a high carbon formation rate. In addition, the oxygen storage capacity (OSCC) of Ni/CeO_2_-500-35 and Ni/CeO_2_-D-35 was tested by CO pulse. The high oxygen storage capacity contributed to the higher carbon removal capacity of Ni/CeO_2_-D-35, and then reduced the rate of carbon deposition. So, the amount of carbon deposition on Ni/CeO_2_-D-35 was less than that on Ni/CeO_2_-500-35. In addition, the high degree of carbon deposition further hindered the removal of carbon on Ni/CeO_2_-500-35. Therefore, a large amount of carbon deposition and the high degree of carbon deposition synergistically led to the catalytic stability of Ni/CeO_2_-500-35, which was inferior to that of Ni/CeO_2_-D-35.

## 4. Conclusions

In conclusion, CeO_2_ with spheres were prepared for supported nickel and used in GSR. The catalytic performance of Ni/CeO_2_-D was significantly improved by direct reduction without the thermal calcination step. Further physicochemical characterization was applied to reveal the reasons for excellent catalytic performance of Ni/CeO_2_-D. The results of the characterization showed that direct hydrogen reduction treatment favored the acquisition of highly dispersed Ni and small-sized Ni nanoparticles. The relatively weak Ni–CeO_2_ interaction facilitated the reduction of Ni nanoparticles of Ni/CeO_2_-D. As a result, the improved surface area of the active Ni^0^ was obtained for Ni/CeO_2_-D. In addition, the direct hydrogen reduction treatment resulted in more Ce^4+^ reduced to Ce^3+^, which was accompanied by the generation of more oxygen vacancies. The high concentration of oxygen vacancies not only inhibited the growth of nickel nanoparticles size by anchoring the nickel nanoparticles, but it also promoted the water–gas shift reaction. Therefore, the improved surface area of the active Ni^0^ and the high concentration of oxygen vacancies synergistically improved the catalytic activity. In addition, high oxygen storage capacity accelerated the removal of carbon deposition and prolonged the catalyst stability.

## Figures and Tables

**Table 1:** Textural parameters and lattice parameters of support and catalysts.

Catalysts	Ni Content (wt.%)	Surface Area (m^2^/g)	Pore Volume (cc/g)	BJH Pore Size (nm)	CeO_2_ Lattice Parameter (Å)
CeO_2_	-	153.8	0.32	3.9	5.412
Ni/CeO_2_-500	7.05%	110.8	0.27	4.3	5.406
Ni/CeO_2_-D	4.15%	137.9	0.29	4.1	5.409

**Table 2:** Physical and chemical properties of the Ni/CeO_2_ catalysts: XPS data, Raman data, and CO-OSC/OSCC.

Catalyst	Ce^3+^/(Ce^3+^ + Ce^4+^) (%)	Ni^0^/(Ni^0^ + Ni^2+^) (%)	I_D_/I_F2g_	CO-OSC/OSCC (mmolOg^−1^_cat_)
Ni/CeO_2_-500	18.05	24.57	0.216	3.79/40.27
Ni/CeO_2_-D	18.81	25.01	0.268	3.90/43.59

**Table 3:** Physical and chemical properties of the Ni/CeO_2_ catalysts by H_2_ chemisorption.

Catalyst	Metal Dispersion ^a^ (%)	Active Nickel Surface Areas (m^2^/g_Ni_) ^a^	TOF _(_s^−1^_)_ ^b^
Ni/CeO_2_-500	7.65	10.21	7.45
Ni/CeO_2_-D	7.87	10.35	7.63

^a^ Determined by H_2_ chemisorption ^b^ mole _Glycerol_. suf Ni^−^^1.^

**Table 4:** The comparison table with the reported results on the H_2_ production and steam reforming of glycerol is shown below.

Catalysts	Temperature (°C)	nH_2_O/Gly	WHSV/ mL/g_cat_/h	Initial Conversion/ H_2_ Selectivity/%	Conversion/H_2_ Selectivity after 16 h/%	H_2_ Yield (mol/molGly)	Ref.
Ni/Ce	475	9	28,000	95/93	95/93	5.4/5.4	present work
Ni/CeAl	700	9	42,000	80/78	89/71	-	[27]
Ni/LaAl	600	9	50,000	93/96	80/86	3/3	[41]
Ni-Cr/SBA-15	600	6	-	100/65	95/55	-	[42]
Ni/Al	600	20	50,000	90/60	52/80	3.35/0.65	[43]
10Ni/Al	600	-	-	100/92	100/82	-	[44]
Rh/MgAl	600	9	50,000	55/88	45/78	2.9/2.4	[45]
10Ni/Si	600	-	21,300	89/88	70/81	3.1/2.5	[46]

**Table 5:** Quantification analysis of carbon deposition on spent catalysts.

Catalyst	Weight Loss (%)	Carbon/Glycerol (mmol/mol)	Carbon Formation Rate (mmol/gcat/h)	C Balance
Ni/CeO_2_-500	14.1	15.2	0.39	99.7%
Ni/CeO_2_-D	11.2	11.8	0.3	99.8%

## Data Availability

All data used to support the findings of this study are included within the article.
